# Determining the Rate-Limiting Step for Light-Responsive Redox Regulation in Chloroplasts

**DOI:** 10.3390/antiox7110153

**Published:** 2018-10-31

**Authors:** Keisuke Yoshida, Toru Hisabori

**Affiliations:** Laboratory for Chemistry and Life Science, Institute of Innovative Research, Tokyo Institute of Technology, Nagatsuta 4259-R1-8, Midori-ku, Yokohama 226-8503, Japan

**Keywords:** redox regulation, thioredoxin, ferredoxin-thioredoxin reductase, chloroplast

## Abstract

Thiol-based redox regulation ensures light-responsive control of chloroplast functions. Light-derived signal is transferred in the form of reducing power from the photosynthetic electron transport chain to several redox-sensitive target proteins. Two types of protein, ferredoxin-thioredoxin reductase (FTR) and thioredoxin (Trx), are well recognized as the mediators of reducing power. However, it remains unclear which step in a series of redox-relay reactions is the critical bottleneck for determining the rate of target protein reduction. To address this, the redox behaviors of FTR, Trx, and target proteins were extensively characterized in vitro and in vivo. The FTR/Trx redox cascade was reconstituted in vitro using recombinant proteins from *Arabidopsis*. On the basis of this assay, we found that the FTR catalytic subunit and *f*-type Trx are rapidly reduced after the drive of reducing power transfer, irrespective of the presence or absence of their downstream target proteins. By contrast, three target proteins, fructose 1,6-bisphosphatase (FBPase), sedoheptulose 1,7-bisphosphatase (SBPase), and Rubisco activase (RCA) showed different reduction patterns; in particular, SBPase was reduced at a low rate. The in vivo study using *Arabidopsis* plants showed that the Trx family is commonly and rapidly reduced upon high light irradiation, whereas FBPase, SBPase, and RCA are differentially and slowly reduced. Both of these biochemical and physiological findings suggest that reducing power transfer from Trx to its target proteins is a rate-limiting step for chloroplast redox regulation, conferring distinct light-responsive redox behaviors on each of the targets.

## 1. Introduction

Thiol-based redox regulation is a post-translational mechanism for the control of enzymatic activity through modification of redox-active Cys residues on the target protein (e.g., formation/cleavage of disulfide bonds). A small protein, thioredoxin (Trx), serves as a key factor in redox regulation. Trx has a highly conserved amino acid sequence of WCGPC at its active site, allowing it to reduce disulfide bonds of its target proteins through the dithiol-disulfide exchange reaction. Trx was first identified in *Escherichia coli* in 1964 as a ribonucleotide reductase cofactor [1]. It is now known that the Trx-dependent redox regulation system is ubiquitously preserved in all kingdoms of life and adjusts biological functions in response to changes in local redox environments.

In plant chloroplasts, the redox regulation system is unique in terms of linking to the excitation of photosynthetic electron transport and, thereby, light. Upon illumination, a part of the reducing power generated from photochemical reactions is transmitted from the photosynthetic electron transport chain to the ferredoxin-thioredoxin reductase (FTR)/Trx redox cascade [2,3,4]. FTR is a soluble [4Fe-4S] protein and acts as a signaling hub linking photosynthetically reduced ferredoxin (Fd) to Trx [5,6]. A reduced form of Trx, in turn, transfers reducing power to a specific set of redox-sensitive target proteins in chloroplasts. Some Calvin-Benson cycle enzymes, including fructose 1,6-bisphosphatase (FBPase) and sedoheptulose 1,7-bisphosphatase (SBPase), are well-known targets; they are activated upon reduction [7]. Consequently, the FTR/Trx redox cascade plays a key role in switching on photosynthetic carbon metabolism in a light-coordinated manner. This is a canonical pathway for chloroplast redox regulation, pioneered by Buchanan and colleagues [2,3].

Since the turn of the century, our understanding of chloroplast redox regulation has been increasingly extended. Progress in plant genomic and proteomic studies has revealed a large number of factors that constitute the redox regulation system in chloroplasts. For example, five Trx subtypes (*f*-, *m*-, *x*-, *y*-, and *z*-type) have been identified in chloroplasts [8,9]. Despite a common active site motif and structure, they show different biochemical characteristics, such as their protein surface charges and midpoint redox potentials [10,11,12]. Furthermore, several hundreds of chloroplast proteins, which are involved in a broad spectrum of biological processes, have been suggested as potential targets of Trx [13,14]. These emerging data indicate that the redox regulation system is highly organized in chloroplasts, flexibly controlling an array of functions. A number of studies have been directed toward revealing the molecular basis and physiological significance of the redox-based regulatory network in chloroplasts; however, many aspects remain to be elucidated (for recent reviews, see [15,16]).

As described above, the FTR/Trx redox cascade acts as a pivotal pathway in supporting light-responsive redox regulation in chloroplasts. Although the regulatory system itself has been firmly established, the rate-limiting step in the redox-relay process remains elusive. Answering this question is important both to the field of basic biology and biotechnology of plants, because the rate of target protein reduction is linked directly to the activating kinetics of key photosynthetic reactions (including ATP synthesis and the Calvin–Benson cycle) and may ultimately impact on overall photosynthetic performance. To this end, the redox behaviors of FTR, Trx, and target proteins need to be characterized individually. In this study, we addressed this issue from both in vitro and in vivo standpoints, allowing us to identify the bottleneck in light-responsive redox regulation in chloroplasts. The data from this study provide an insight into the working dynamics of chloroplast redox regulation under varying light environments.

## 2. Materials and Methods

### 2.1. Preparation of *Arabidopsis* Recombinant Proteins

All expression plasmids used in this work were constructed during previous studies [12,17,18]. Each expression plasmid was transformed into *E. coli* strain BL21 (DE3) (for ferredoxin-NADP^+^ reductase (FNR), FTR heterodimer, Trx-*f*1, Trx-*m*1, Trx-*x*, Trx-*y*2, Trx-*z*, FBPase, and SBPase) or Rosetta (DE3) pLysS (for Rubisco activase (RCA; redox-sensitive isoform)). Transformed cells were cultured at 37 °C. The expression was induced by the addition of 0.5 mM isopropyl-1-thio-β-d-galactopyranoside followed by overnight culture at 21 °C. Cells were disrupted by sonication. After centrifugation (125,000× *g* for 40 min), the resulting supernatant was used to purify the protein. All recombinant proteins contained no affinity tag, and they were purified by a combination of anion-exchange chromatography, hydrophobic-interaction chromatography, and size-exclusion chromatography, as described previously [12,17]. The protein concentration was determined with a BCA protein assay (Pierce, Rockford, USA).

### 2.2. Reconstitution of FTR/Trx Redox Cascade and Determination of Protein Redox State In Vitro

The FTR/Trx redox cascade was reconstituted in vitro as previously described [17] with modifications. All proteins (FNR, Fd (from spinach; Sigma-Aldrich, St. Louis, MO, USA), FTR heterodimer, the indicated Trx isoform, and the indicated target protein) were incubated at 2 μM in medium containing 50 mM Tris-HCl (pH 7.5 or 8.2) and 50 mM NaCl. Reducing power-transferring reactions were initiated by the addition of 1 mM nicotinamide adenine dinucleotide phosphate (NADPH). This assay was performed at 25 °C. After the reaction, the redox state of the protein was determined by identifying the thiol status with the use of thiol-modifying reagent 4-acetamido-4′-maleimidylstilbene-2,2′-disulfonate (AMS) as previously described [17].

### 2.3. Plant Materials, Growth Conditions, and High Light Treatments

*Arabidopsis thaliana* wild-type plants (Col-0) were grown in soil in a controlled growth chamber (light intensity, 70–80 µmol photons m^−2^ s^−1^; temperature, 22 °C; relative humidity, 60%; 16 h day/8 h night) for four weeks. For high light (HL) treatments, plants were placed in the dark for 8 h and then irradiated at 650–700 μmol photons m^−2^ s^−1^ for the time period indicated.

### 2.4. Measurement of Photosynthetic Electron Transport Rate

Chlorophyll fluorescence and absorbance change at 830 nm were measured simultaneously using a Dual-PAM-100 (Walz, Effeltrich, Germany) with the intact leaves. A saturating pulse of red light (800 ms, >5000 μmol photons m^−2^ s^−1^, 635 nm) was applied to calculate the quantum yields of photosystem (PS)II (Y (II)) and PSI (Y (I)). Relative electron transport rates of PSII (ETR II) and PSI (ETR I) were calculated as Y (II) × light intensity and Y (I) × light intensity, respectively.

### 2.5. Determination of Protein Redox State In Vivo

Plants were frozen using liquid nitrogen, and the redox state of the protein was determined as previously described [19].

## 3. Results

### 3.1. The In Vitro Biochemical Study

Firstly, comparative characterization of the reduction kinetics of FTR, Trx, and target proteins was attempted using an in vitro biochemical procedure. The *Arabidopsis* FTR/Trx redox cascade was reconstituted as previously described [17] with modifications; in this study, all proteins were incubated at an equimolar concentration (2 μM each). These changes to the procedure were introduced in order to determine the bottleneck in reducing power transfer at the protein molecule level. The redox state of the protein was identified using the thiol-modifying reagent AMS. AMS binds to free thiols and then lowers the protein mobility on Sodium dodecyl sulfate polyacrylamide gel electrophoresis. (SDS-PAGE), allowing the determination of redox state as a band shift [20].

Three stromal proteins were prepared for use as targets; these included FBPase, SBPase, and RCA. Previous reports have noted that Trx-*f* is the most effective Trx subtype to reduce these targets in the presence of the chemical reductant dithiothreitol (DTT) [10,12,21]. As a preliminary test, whether the preference for Trx-*f* is also true in the reconstituted FTR/Trx system was investigated (Figure S1). FBPase, SBPase, and RCA were efficiently shifted from oxidized to reduced forms by Trx-*f*. These results were obtained both at pH 7.5 and 8.2. On the basis of these data, Trx-*f* was chosen for the following biochemical assays.

**Figure 1:**
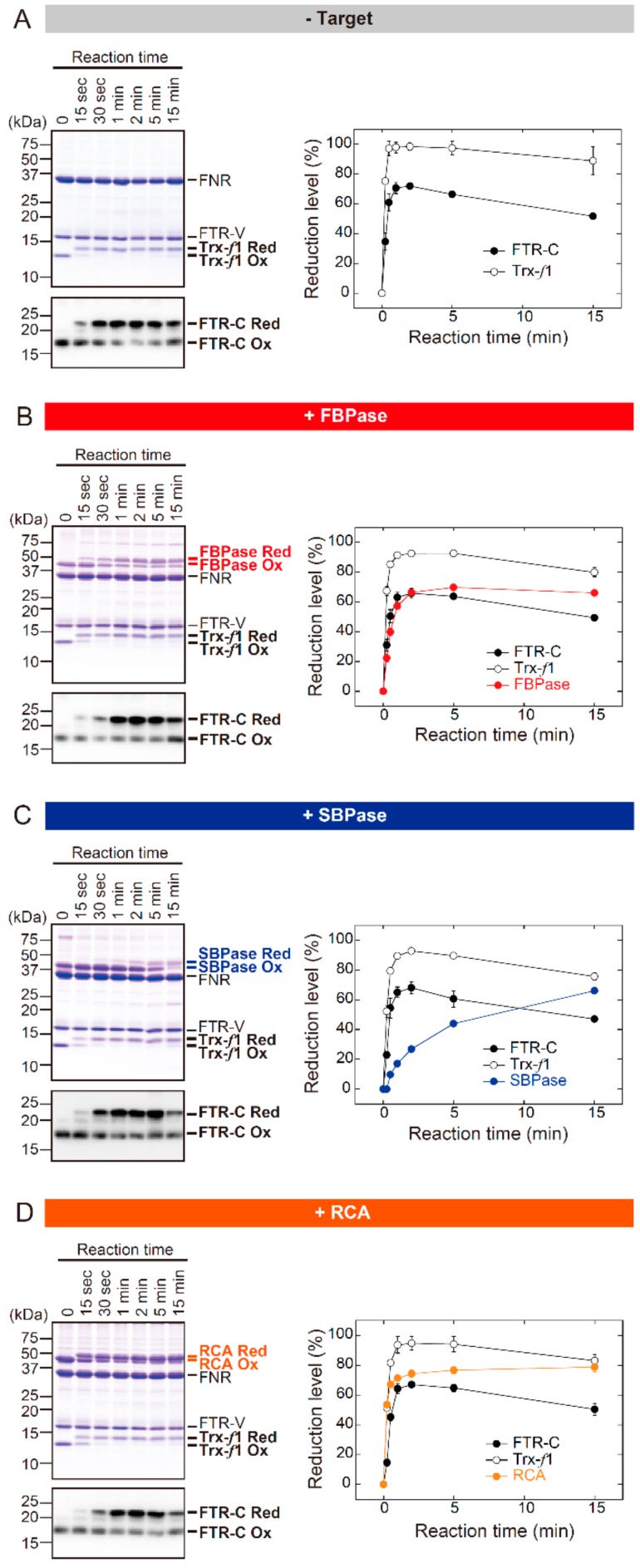
The in vitro redox responses of FTR-C, Trx-*f*1, and target proteins from *Arabidopsis* (pH 7.5). (**A**) Experiments were performed under target-free conditions. (**B**–**D**) FBPase (**B**), SBPase (**C**), or RCA (**D**) was used as the target. Each of the oxidized proteins (2 μM) was incubated with 1 mM NADPH, 2 μM FNR and 2 μM Fd for the indicated time period. Assays were performed at pH 7.5. Following the reaction, proteins were labeled with AMS and subjected to non-reducing SDS-PAGE. Proteins were then stained with Coomassie Brilliant Blue R-250 (CBB). FTR-C was detected by immunoblotting, as its CBB-derived signal was low. The reduction level of each protein was calculated as the ratio of the reduced form to the total. Values represent the mean ± SD (*n* = 3). FTR-V, FTR variable subunit; Red, reduced form; Ox, oxidized form. FTR-C: FTR catalytic subunit; FBPase: Fructose 1,6-bisphosphatase; SBPase: Sedoheptulose 1,7-bisphosphatase; RCA: Rubisco activase; NADPH: Nicotinamide adenine dinucleotide phosphate; FNR: Ferredoxin-NADP^+^ reductase; AMS: 4-acetamido-4′-maleimidylstilbene-2,2′-disulfonate; SDS-PAGE: Sodium dodecyl sulfate polyacrylamide gel electrophoresis.

Figure 1 shows the time course for redox change in the FTR catalytic subunit (FTR-C), Trx-*f*, and target proteins. These assays were performed at pH 7.5. Under target-free conditions, FTR-C and Trx-*f* were rapidly shifted from oxidized to reduced forms after the onset of the reaction; the redox state of these proteins reached a plateau within 1 min (Figure 1A). Notably, almost identical redox responses of FTR-C and Trx-*f* were observed even in the presence of target proteins (Figure 1B–D). By contrast, largely different reduction rates were seen in the three target proteins. The redox state of FBPase and RCA reached a plateau at 2–5 and 1–2 min, respectively (Figure 1B,D). SBPase was reduced at a fairly low rate; its reduction level was continuously elevated for 15 min (Figure 1C). Taken together, it was suggested that the rate of target protein reduction is primarily determined by the efficiency of reducing power transfer downstream of Trx-*f*. Similar results were observed under different pH conditions (pH 8.2; Figure S2).

### 3.2. The In Vivo Physiological Study

In order to address another major concern, the in vivo redox behaviors of Trx and target proteins were observed. After dark adaptation, *Arabidopsis* plants were transferred to HL conditions (650–700 μmol photons m^−2^ s^−1^). The redox state of several chloroplast proteins (some Trx isoforms and target proteins) was sequentially determined as previously described [19].

**Figure 2:**
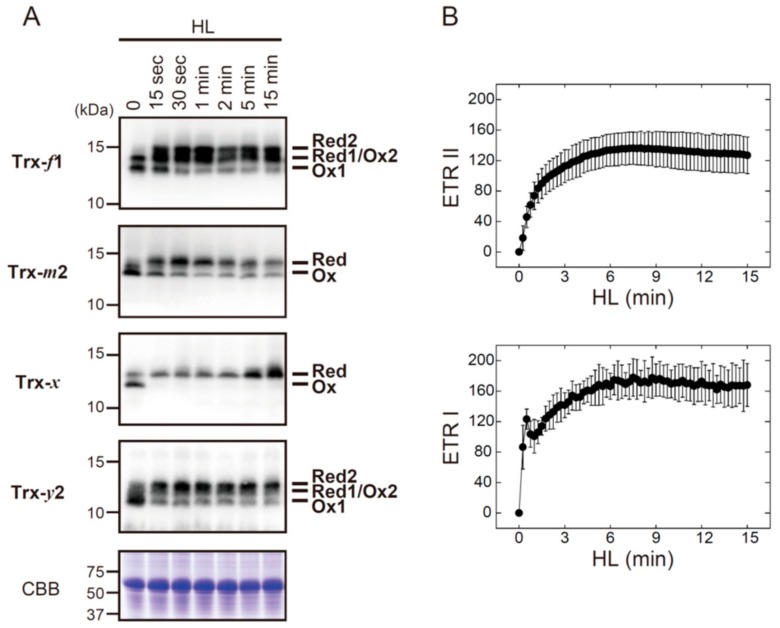
The in vivo redox responses of Trx isoforms and their relationship to photosynthetic electron transport rates in *Arabidopsis*. (**A**) Dark-adapted plants were placed under HL conditions (650–700 μmol photons m^−2^ s^−1^) for the indicated time period. The redox states of Trx-*f*1, Trx-*m*2, Trx-*x*, and Trx-*y*2 were then determined as previously described [19]. As a loading control, Rubisco large subunit was stained with Coomassie Brilliant Blue R-250 (CBB). Red, reduced form; Ox, oxidized form. (**B**) Dark-adapted plants were irradiated by HL, and relative ETR II and ETR I were sequentially measured. Values represent the mean ± SD (*n* = 5). HL: High light; ETR II: Electron transport rates of PSII; ETR I: Electron transport rates of PSI.

Four Trx isoforms (Trx-*f*1, Trx-*m*2, Trx-*x*, and Trx-*y*2) were rapidly converted from oxidized to reduced forms after exposure to HL (Figure 2A). Their redox states apparently reached stable levels within 1 min of HL exposure, which was common with all Trx isoforms examined here. For comparison, we analyzed the induction pattern of photosynthetic electron transport (Figure 2B). ETR II and ETR I were gradually elevated upon exposure to HL, and they attained maximal levels at approximately 6 min. These results indicate that Trx can receive reducing power even when the photosynthetic electron transport is not fully activated during dark‒light transitions.

**Figure 3:**
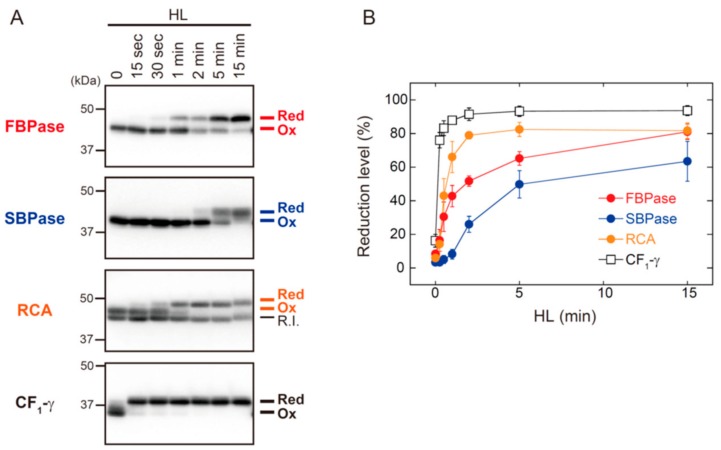
The in vivo redox responses of Trx-targeted proteins in *Arabidopsis*. Dark-adapted plants were placed under HL conditions (650–700 μmol photons m^−2^ s^−1^) for the indicated time period. (**A**) The redox state of FBPase, SBPase, RCA, and ATP synthase CF_1_-γ subunit was determined as previously described [19]. Red, reduced form; R.I., redox-insensitive form of RCA; Ox, oxidized form. (**B**) The reduction level of each protein was calculated as the ratio of the reduced form to the total. Values represent the mean ± SD (*n* = 3).

Figure 3 shows the HL-responsive redox changes in FBPase, SBPase, RCA, and ATP synthase CF_1_-γ subunit. The shift from oxidized to reduced forms of FBPase, SBPase, and RCA occurred with different kinetics. RCA showed the highest reduction rate of these three proteins, but it took approximately 2 min for RCA to reach a highly reduced state. The reduction rates of FBPase, SBPase, and RCA were clearly slower than those of Trx isoforms (for comparison, see Figure 2A). By contrast, CF_1_-γ showed an exceptionally high rate of reduction; CF_1_-γ was almost fully reduced within 15 s of HL exposure. It was thus evident that, even if reducing power is supplied immediately to Trx upon HL exposure, Trx distributes it to each of the targets at largely different rates.

## 4. Discussion and Conclusions

The aim of this study was to determine the rate-limiting step for light-responsive redox regulation in chloroplasts. The in vitro and in vivo studies carried out here led to a consistent conclusion; reducing power transfer from Trx to its target proteins is a critical bottleneck for determining the rate of target protein reduction. The data also highlight that this step confers distinct light-responsive redox behaviors onto each of the targets.

In the reconstituted FTR/Trx system, FTR-C and Trx-*f* exhibited rapid reduction following the drive of reducing power transfer (Figure 1, Figure S2). In agreement with this, the in vivo study revealed that the Trx family was rapidly reduced upon HL irradiation (Figure 2A). These results suggest the close coupling of FTR and Trx to the activation of photosynthetic electron transport. By referring to the data on PSII and PSI quantum yields (Figure 2B), it was shown that Trx actively receives reducing power even during the induction phase of photosynthetic electron transport. It thus seems likely that, together with other systems [22,23], the FTR/Trx system acts as an electron sink during this period. This idea is supported by a previous study showing that, in Trx-*f*-deficient *Arabidopsis*, the induction of photosynthetic electron transport was hampered because of prolonged electron accumulation in the PSI acceptor side [24]. Therefore, an additional role for the FTR/Trx system as an initiator of photosynthesis can be considered. To evaluate its contribution quantitatively, future studies should be directed toward estimating the extent of electron transfer from Fd to the FTR/Trx redox cascade.

In the in vitro experiments, FBPase, SBPase, and RCA showed different rates of reduction; RCA was most rapidly reduced, followed by FBPase and finally SBPase (Figure 1, Figure S2). Because these experiments were performed with a fixed stoichiometry, differential responses of these three proteins must be attributed to the biochemical properties of each molecule, including the midpoint redox potential, the electrostatic charge on the protein surface, and the affinity to Trx-*f*. Different reduction rates for FBPase, SBPase, and RCA were also observed in vivo (Figure 3), but they appeared to be uniformly slower compared with the in vitro responses (Figure 1, Figure S2). This is possibly related to the fact that various target proteins co-exist in chloroplasts in different amounts [12,25]. It should also be noted that the recently identified protein-oxidizing redox cascade works in vivo [18], which may lower the net rate of target protein reduction. Furthermore, the localization of target proteins within chloroplasts seems to be a key intrinsic determinant for their reduction rates, given that the thylakoid membrane-bound ATP synthase CF_1_-γ subunit showed marked rapid reduction upon exposure to HL (Figure 3). In summary, the in vivo reduction kinetics of target proteins is thought to be determined by numerous biochemical and physiological factors in a complex manner. Further studies are needed to clarify the overall mechanisms underlying dynamic and divergent redox behaviors of chloroplast proteins.

## Supplementary Materials

The following are available online at http://www.mdpi.com/2076-3921/7/11/153/s1, Figure S1: Trx selectivity for reducing FBPase, SBPase, and RCA in the reconstituted FTR/Trx system in *Arabidopsis*, Figure S2: The in vitro redox responses of FTR-C, Trx-*f*1, and target proteins from *Arabidopsis* (pH 8.2).
