# Supplementary material for: Determining the Rate-Limiting Step for Light-Responsive Redox Regulation in Chloroplasts
Source: Antioxidants (Basel). 2018 Oct 31;7(11):153. doi: 10.3390/antiox7110153 (PMC6262275; doi:10.3390/antiox7110153)
Supplement: Supplementary file 1 [file antioxidants-07-00153-s001.pdf]

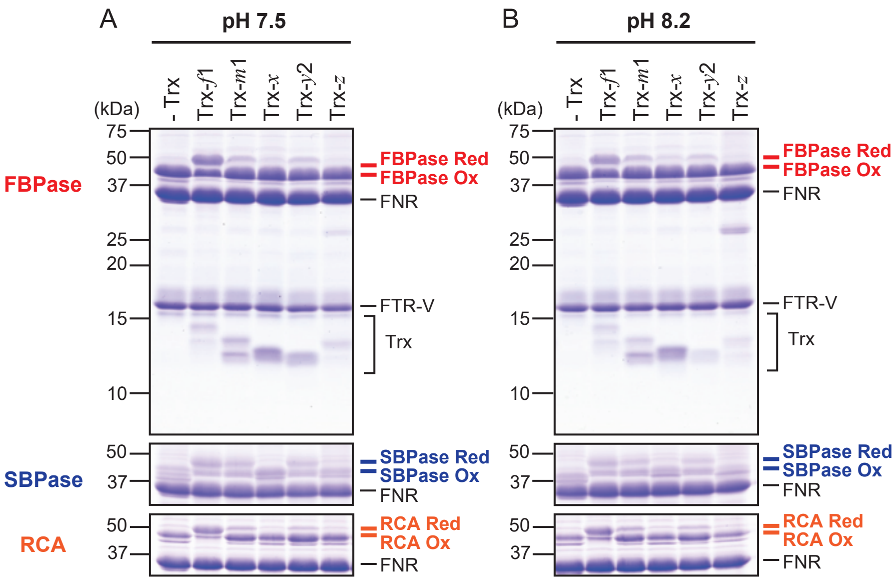

Figure S1. Trx selectivity for reducing FBPase, SBPase, and RCA in the reconstituted FTR/Trx system in *Arabidopsis*. Each of the target proteins (oxidized form; 2 μM) was incubated with 1 mM NADPH, 2 μM FNR, 2 μM Fd, 2 μM FTR, and 2 μM Trx (indicated isoform) for 15 min. Assays were performed at pH 7.5 (A) and 8.2 (B). Following the reaction, proteins were labeled with AMS and subjected to non-reducing SDS-PAGE. Proteins were then stained with Coomassie Brilliant Blue R-250 (CBB). FTR-V, FTR variable subunit; Red, reduced form; Ox, oxidized form.

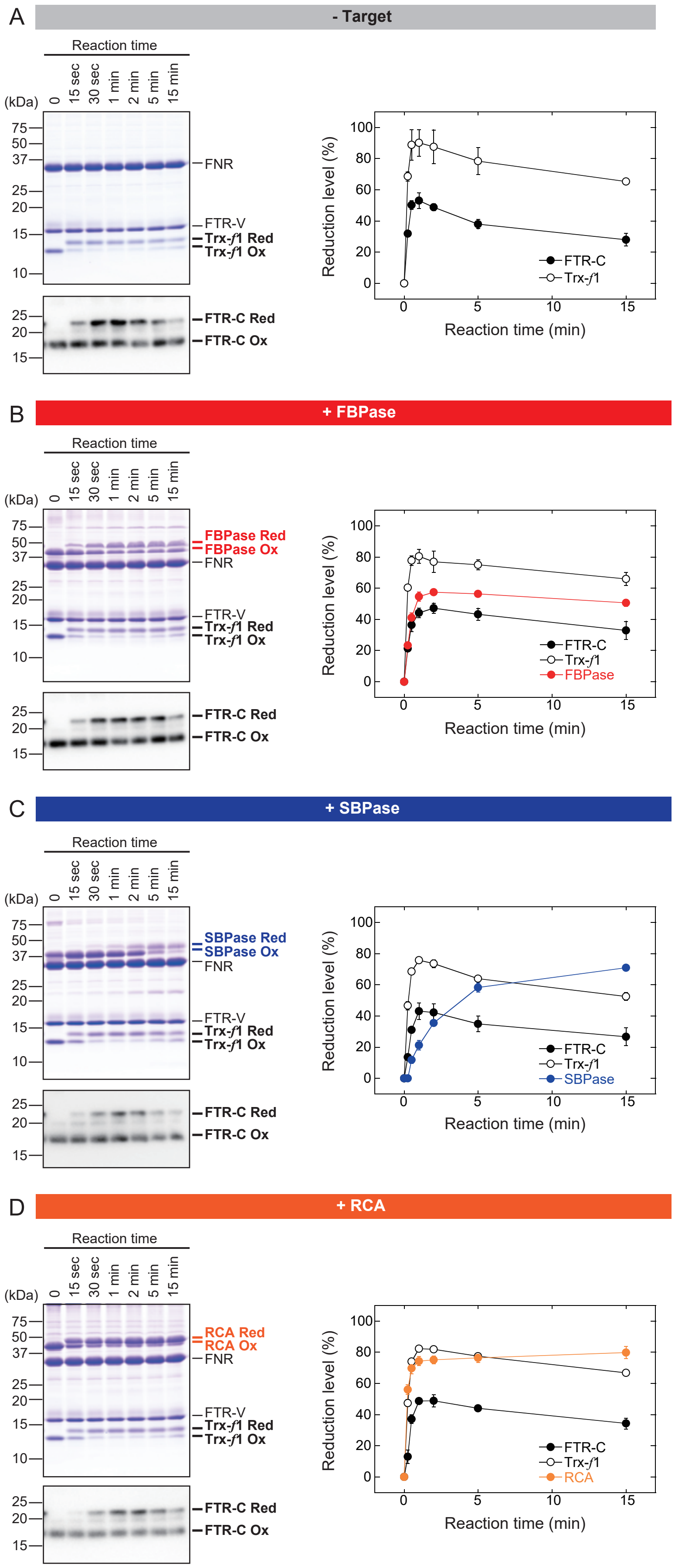

Figure S2. The in vitro redox responses of FTR-C, Trx-*f*1, and target proteins from *Arabidopsis* (pH 8.2). (A) Experiments were performed under target-free conditions. (B-D) FBPase (B), SBPase (C), or RCA (D) was used as the target. Each of the oxidized proteins (2 μM) was incubated with 1 mM NADPH, 2 μM FNR and 2 μM Fd for the indicated time period. Assays were performed at pH 8.2. Following the reaction, proteins were labeled with AMS and subjected to non-reducing SDS-PAGE. Proteins were then stained with Coomassie Brilliant Blue R-250 (CBB). FTR-C was detected by immunoblotting, as its CBB-derived signal was low. The reduction level of each protein was calculated as the ratio of the reduced form to the total. Values represent the mean ± SD (n = 3). FTR-V, FTR variable subunit; Red, reduced form; Ox, oxidized form.
